# Use of etomidate in endotracheal intubations in the emergency room during the COVID-19 pandemic: a randomized clinical trial

**DOI:** 10.1590/acb395724

**Published:** 2024-09-09

**Authors:** Fernando Sabia Tallo, Marcelo Pires-Oliveira, Marianne Yumi Nakai, Lucas Antonio Duarte Nicolau, Jand Venes Rolim Medeiros, Flávia de Sousa Gehrke, Murched Omar Taha, Afonso Caricati-Neto, Francisco Sandro Menezes-Rodrigues, Simone de Campos Vieira Abib

**Affiliations:** 1 Universidade Federal de São Paulo – São Paulo (SP) – Brazil.; 2 Centro Universitário UNIME – Lauro de Freitas (BA) – Brazil.; 3 Irmandade da Santa Casa de Misericórdia de São Paulo – São Paulo (SP) – Brazil.; 4 Universidade Federal do Delta do Parnaíba – Parnaíba (PI) – Brazil.; 5 Centro Universitário Faculdade de Medicina do ABC – Santo André (SP) – Brazil.

**Keywords:** Etomidate, Intubation, Mortality, Anesthesia, General

## Abstract

**Purpose:**

Shock, cardiovascular problems, and respiratory failure constitute the main causes of death in patients cared in medical emergency rooms. Patients commonly require orotracheal intubation (OTI), a fact that has been intensified by diseases that generate important and fatal hemodynamic and respiratory problems in the affected patient.

**Methods:**

Although etomidate (ETO) is a highly used anesthetic for OTI, its use remains controversial in several scenarios. Some studies refer to an increase in mortality with its use in critically patients, while others do not refer to a difference. Therefore, we evaluated the mortality of patients submitted to OTI in the public hospital of a public federal university, with the use of ETO and other sedative-hypnotic drugs used in the induction of the performance of OTI, with the in-hospital mortality of patients cared in hospital.

**Results:**

The results demonstrate that the use of ETO as a hypnotic for OTI in the emergency room is not associated with a significant difference in morbidity or early mortality, within 30 days of hospitalization, compared with other hypnotics.

**Conclusions:**

There was no difference in mortality between patients intubated in the emergency department who used ETO and those who used non-ETO hypnotic within 72 hours and 30 days.

## Introduction

Orotracheal intubation (OTI) is a common emergency department procedure in medium and large hospitals. It is of a highly technical nature and of great relevance for the treatment of a wide range of patients, such as those affected by car accident trauma, stroke, acute myocardial infarction, pneumonia or COVID-19^1^^–^ 3. Literature data describe important differences regarding adverse reactions and complications arising from the anesthetic drug used, such as, for example, nausea, vomiting, peripheral vasodilation, myocardial depression, increased intracranial pressure, and cardiorespiratory arrest^3^.

Several drugs are used as adjuvants in this high-risk procedure to minimize orotracheal injuries, discomfort to the patient, and damage to the professionals involved in performing it. These include neuromuscular junction blockers, such as suxamethonium and rocuronium; antihistamines; antiemetics; benzodiazepines (midazolam); opioids, such as fentanyl; and general anesthetics, such as propofol, ketamine, and etomidate. These latter are the most used intravenous anesthetic agents used for induction of general anesthesia prior to rapid sequence intubation in critically ill patients^4^.

The choice of an appropriate anesthetic requires evaluation of several physiological and pathological variables involving cardiovascular, respiratory, and neurological system function in the patient’s current clinical conditions. A recent multicenter and cross-sectional study with critically ill patients treated in an intensive care unit (ICU) in the United States of America has shown that there are significant differences in the choice of medications that are often incorrect and inappropriate when hemodynamic parameters of the patients are critically analyzed^5^.

Physicians working in the emergency service prefer to use propofol when performing rapid sequence intubation of trauma patients treated by an anesthesiologist, while emergency patients are treated with ketamine or etomidate in unstable patients by emergency physicians^6^^–^ 8. Each of these anesthetic agents has advantages and disadvantages, and one is not necessarily superior to the other. Propofol (2,6-Bis [1-methylethyl] phenol), a sedative and hypnotic, can lower systemic blood pressure, especially in hypovolemic patients, the elderly, and those with reduced left ventricular function^6^^–^ 8.

In trauma patients, propofol use was associated with more hypotension than non-propofol agents without long-term consequences, and the occurrence of hypotension may depend on the dose of propofol used^8^. Ketamine (2-[2-Chlorophenyl]-2-[methylamino]-cyclohexanone hydrochloride), a sedative, analgesic, and N-Methyl-d-aspartate receptor antagonist, has a favorable hemodynamic effect, but it may cause hypotension in case of catecholamine depletion or direct negative inotrope effect^9^^,^ 10. Etomidate (1-[1-phenylethyl]-1-himidazole-5-carboxylic acid ethyl ester), a sedative and hypnotic, also has a favorable hemodynamic profile, but it has been associated with a high rate of transient adrenal insufficiency and mortality, especially in patients with sepsis^11^^–^ 14.

Eventually, any drug, when properly administered, may be adequate among the patients undergoing rapid sequence intubation in the emergency department for various medical and surgical emergencies. The choice of anesthetic agent influences the results when adjusted for the severity of the disease^11^^,^ 12. However, there was great variability in the use of these drugs, as recently demonstrated in children with trauma; propofol was commonly used and potentially associated with worse outcomes^15^.

Therefore, the aim of this study was to evaluate the association between the use of any of these induction agents and ICU and hospital outcomes (mortality and length of stay). Our hypothesis was that the hospital mortality would be lower with propofol or etomidate (ETO) than with ketamine when used as an induction agent for intubation of critically ill patients in the ICU.

## Methods

This study was a randomized, multicentric clinical trial in 11 emergency departments to compare the use of ETO with non-etomidate anesthesia in intubated emergency room adult patients. Eligible patients were all adults (≥ 18 years) who required emergency endotracheal intubation (EEI) between June 2020 and December 2021 in participant research centers. Only patients that required EEI in the emergency room were deemed eligible. The exclusion criteria were intubation due to cardiac arrest and allergies/hypersensitivity to fentanyl, midazolam, ketamine, propofol, or ETO. A simple computer-generated randomization was performed to assign patients to ETO or non-ETO endotracheal intubation groups.

All patients received standard ICU monitoring, including noninvasive blood pressure (BP), continuous cardiac monitoring, and real-time pulse oximetry. BP was measured each minute during intubation and each 15 minutes post-intubation with an appropriately sized sphygmomanometer on an upper limb. Noninvasive BP measurements were used for primary analysis. After intubation, all patients received standard sedative anesthesia with 0.2–0.5 μg/kg/h midazolam and 2–5 μg/kg/h fentanyl in the emergency room while awaiting transfer.

For each patient, the registry included:

- Drugs and doses used;
- Patient identification data;
- Previous airway evaluation;
- Hemodynamic data, pre- and post-intubation;
- Comorbidities;
- Airway access indication;
- Body mass index;
- Intubation-related complications;
- Post-intubation patient destination;
- P1;
- Mechanical ventilation duration in the emergency room;
- Experience and qualifications of the professional who performed the intubation – those who did not perform on average at least two orotracheal intubation/week for the last year and was not a medical resident were considered inexperienced;
- Results of a 30-day follow-up.

A logistic regression model was used to analyze how certain factors influenced the immediate or post-30-day death rate. Numeric and continuous variables were described as mean ± standard deviation, unless otherwise specified. The categorical and ordinal variables were described with frequency and total count.

Univariate analyses were used to evaluate differences between the randomized groups. The normality distribution was tested in all continuous variables with histogram, kurtosis, and skewness. The T-test was used for continuous variables, and Fisher’s exact test was used for ordinal/nominal variables.

For subgroups analysis, the relationship between the exposure and the outcomes was evaluated with multiple logistic regression. To adjust the results, the regression model included all the unbalanced variables, potential confounders, and effect modifiers. Univariate analyses were used to evaluate unbalances between the subgroups in baseline characteristics. The selection of covariates for inclusion in the best model was performed as previously described^16^.

## Results

**Figure 1:**
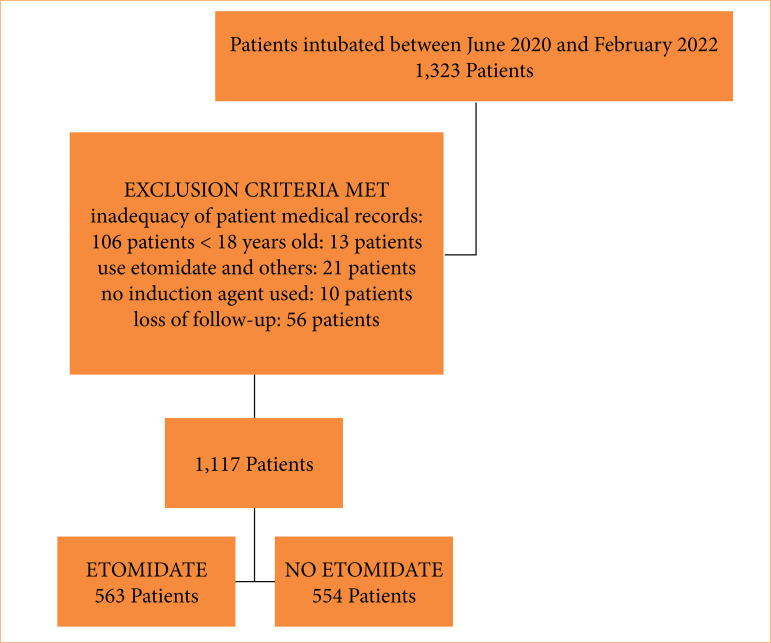
Flowchart of the research sample analyzed.

Source: Elaborated by the authors.

A total of 1,323 severe patients required EEI between February 1st, 2020, and January 20th, 2022, and was considered for inclusion in this study. Figure 1 shows the patient flow diagram for this study. Out of 1,323 patients, 106 were excluded due to inadequate filling of research forms; 56 others were lost during the 30-day follow-up, and 44 were excluded due to use of multiple or non-hypnotic drugs during the EEI procedure, resulting in 563 patients in the ETO group and 554 in the control, non-ETO group.

Table 1 shows the demographic and clinical basal characteristics of the 1,117 analyzed patients. Table 2 shows that, in patients followed for 30 days, the mortality rate was similar in the ETO and non-ETO groups (399 vs. 404, *p* = 0.41). In addition, hospital mechanical ventilation time (6.7 vs. 7.1 days, *p* = 0.56), and mechanical ventilation time (21 h vs. 19.9 h, *p* = 0.39) were similar in ETO and non-ETO groups.

**Table 1:** Patient characteristics by induction agent, etomidate (ETO) or non-etomidate (Non-ETO) groups.

Characteristics	ETO (n = 563)	Non-ETO (n = 554)
**Demographics**		
Gender (female). n (%)	270 (50.4)	266 (49.6)
Age (years old), median	66.6	65.3
**Patient characteristics in emergency department**		
Systolic blood pressure (mmHg)	112.5 ± 17.1	112.9 ± 18.0
Diastolic blood pressure (mmHg)	71.9 ± 11.0	73.5 ± 10.3
SatO_2_	81.2 ± 9.8	79.9 ± 11.2
Respiratory rate	28.5 ± 5.7	28.5 ± 4.4
Heart rate	116.9 ± 13.5	113.5 ± 12.0
**COVID-19**	497 (88.3%)	480 (86.6٪)
**Comorbidities**		
Diabetes *mellitus*	200 (35.5%)	248 (44.8٪)
Systemic arterial hypertension	304 (54.0%)	340 (61.4٪)
> 3	148 (26.3%)	166 (30.0٪)
Body mass index > 30	139 (24.7%)	147 (26.5٪)
**Performer of OTI**		
P1	215 (38.2%)	259 (46.8٪)
P2	100 (17.8%)	89 (16.1٪)
P3	154 (27.4%)	96 (17.3٪)
P4	83 (14.7%)	109 (19.7٪)
P5	11 (2.0%)	1 (0.2٪)
**Sepsis**	142 (25.2%)	131 (23.6٪)

P1: OTI performer with < 1 year of experience; P2: OTI performer with > 1 year of experience; P3: specialist doctor OTI performer; P4: medical residency trainee OTI performer; P5: anesthesiologist OTI performer (> 2 OTI per week for two years). Source: Elaborated by the authors.

**Table 2:** Primary and secondary outcomes according to induction agent, etomidate (ETO) or non-etomidate (non-ETO).

Characteristics	ETO	Non-ETO	*p*–value
Mechanical ventilation time in emergency department (hours; median and interquartile range)	18.0 (3.0–33.0)	14.0 (3.0–30.0)	0.35
Total duration of mechanical ventilation in hospital (days; median and interquartile range)	6.0 (4.0–13.0)	7.0 (4.0–15.0)	0.10
Destination intensive care unit	413 (73.4%)	405 (73.1٪)	0.92

Source: Elaborated by the authors.

**Figure 2:**
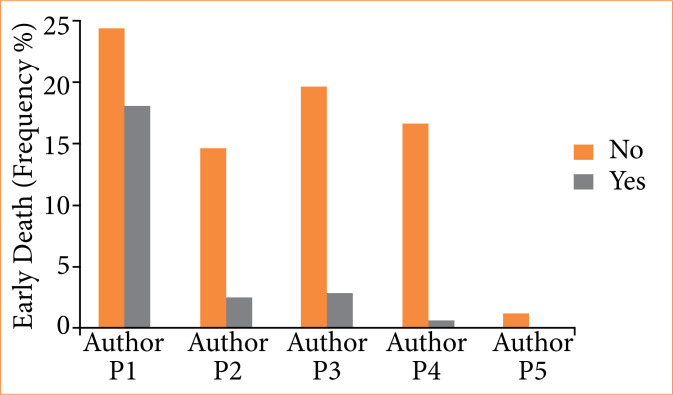
Relationship between the professional training and experience of the medical professional responsible for performing orotracheal intubation (P1, P2, P3, P4, and P5) and the early death (frequency %).

P1: orotracheal intubation performer with < 1 year of experience; P2: orotracheal intubation performer with > 1 year of experience; P3: specialist doctor orotracheal intubation performer; P4: medical residency trainee orotracheal intubation performer; P5: anesthesiologist orotracheal intubation performer (> two orotracheal intubation per week for two years). Source: Elaborated by the authors.

The use of ETO or another hypnotic (143 vs. 121, p = 0.14), as well as the use or not of opioids (135 vs. 128, *p* = 0.49) in OTI, had no effect on early mortality (Table 3). Among patient characteristics, male sex and comorbidities were correlated with higher mortality. OTIs performed by P1 operators (< 1 year of experience) had higher mortality than those performed by more experienced operators (Table 3 and Fig. 2).

**Table 3:** Relative risk of an early death outcome according to patient characteristics or induction agent used in orotracheal intubation.

Characteristics	Yes (%)	No (%)	*p*-value	Relative risk (CI50)
Etomidate	143 (25.4)	120 (21.7)	0.16	1.17 (0.95–1.45)
Male sex	183 (31.6)	79 (14.7)	**< 0.0001**	**2.14** **(1.69–2.72)**
COVID-19	228 (23.3)	35 (25.0)	0.67	0.93 (0.70–1.28)
Any comorbidities	254 (26.1)	9 (6.2)	**< 0.0001**	**4.17** **(2.26–7.92)**
Diabetes *mellitus*	131 (29.2)	132 (20.0)	**0.0005**	**1.46** **(1.18–1.80)**
Hypertension	157 (24.4)	106 (22.4)	0.48	1.09 (0.88–1.35)
Body mass index > 40	44 (41.9)	219 (21.6)	**< 0.0001**	**1.94** **(1.48–2.46)**
P1	201 (42.4)	62 (9.6)	**< 0.0001**	**4.40** **(3.40–5.70)**
Fentanyl	135 (23.6)	128 (23.4)	0.94	1.01 (0.82–1.25)
Difficult airway	31 (32.0)	218 (22.3)	**0.043**	**1.43** **(1.03–1.92)**

P1: orotracheal intubation performer with < 1 year of experience. Source: Elaborated by the authors.

**Figure 3:**
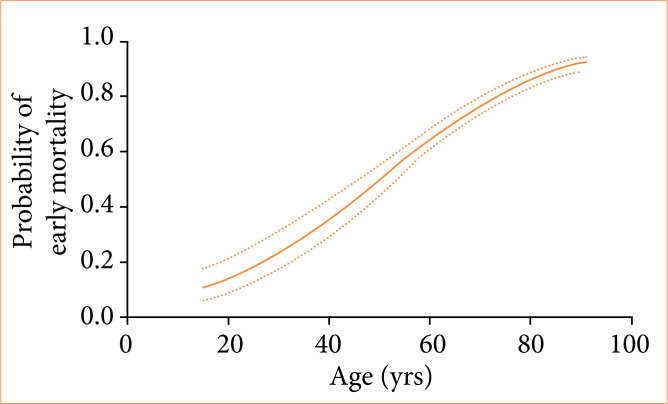
Relationship between the age of patients and the probability of their premature death within a 30-day period.

Source: Elaborated by the authors.

**Figure 4:**
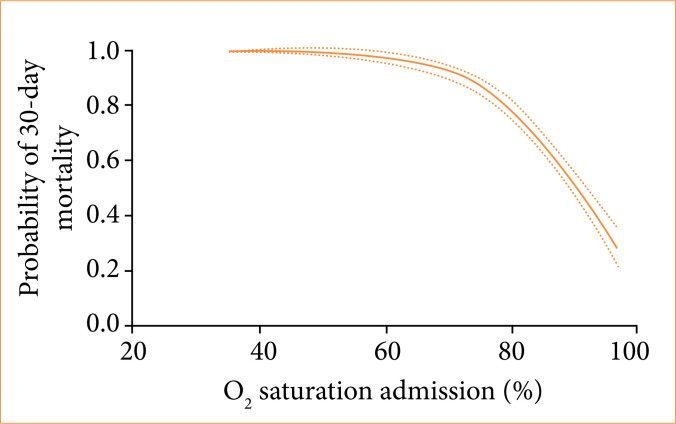
Relationship between O_2_ saturation at patient admission and the probability of mortality within a 30-day period.

Source: Elaborated by the authors.

**Figure 5:**
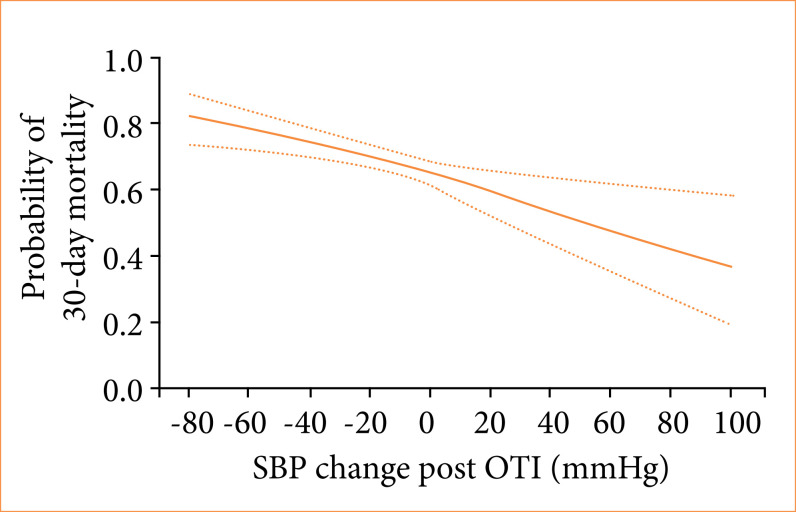
Relationship between systolic blood pressure (SBP) change post orotracheal intubation (OTI) and the probability of mortality within a 30-day period.

Source: Elaborated by the authors.

**Figure 6:**
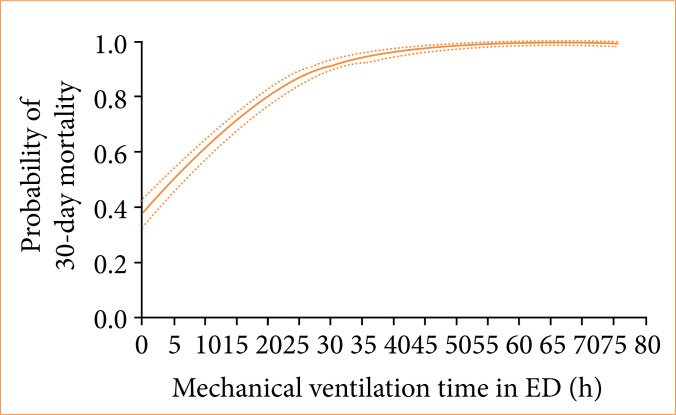
Relationship between mechanical ventilation time in the emergency department (ED) in hours and the probability of mortality within a 30-day period.

Source: Elaborated by the authors.

Higher mortality rates within 30 days of follow-up were seen among patients with COVID-19 (73.7% vs. 61.4%, *p* = 0.002), diabetes *mellitus* (79.2% vs. 67.9%, *p* < 0.001), body mass index > 40 (81.5% vs 71.3%, *p* = 0.026), chronic obstructive pulmonary disease (85.8% vs 68.6%, *p* < 0.001), cardiac heart failure (94.7% vs. 70.6%, *p* < 0.001) and chronic hypertension (75.2% vs 68.7%, *p* = 0.015) (Table 4). There was an increase in the probability of death within 30 days for patients intubated by P1 operators (81.3% vs. 70.3%, *p* = 0.001) and those who suffered complications during the OTI (89.6% vs. 55.8%, *p* < 0.001) (Table 4). The variables correlated with increases in 30-day mortality in our univariate analysis model were patient age (Fig. 3), low O_2_ saturation at admission (Fig. 4) or complications related to the OTI procedure, particularly the greater the difference in systolic blood pressure (SBP) before OTI and after OTI, the greater the early mortality (Fig. 5). In these cases of high SBP increases, the use of opioids decreased early mortality. Longer lengths of mechanical ventilation in the emergency room were also associated with higher 30-day mortality (Fig. 6).

**Table 4:** Relative risk of death within 30 days post-orotracheal intubation outcome according to patient characteristics or induction agent used in orotracheal intubation.

Characteristics	Yes (%)	No (%)	*p*-value	Relative risk (CI50)
Etomidate	396 (70.6)	400 (72.7)	0.46	0.97 (0.90–1.04)
Male sex	410 (70.9)	386 (72.4)	0.59	0.98 (0.91–1.06)
COVID-19	710 (73.1)	86 (61.4)	**0.0050**	**1.19** **(1.05–1.38)**
Any comorbidities	732 (75.7)	64 (44.4)	**< 0.0001**	**1.70** **(1.43–2.08)**
Diabetes *mellitus*	343 (76.6)	453 (68.3)	**0.0028**	**1.12** **(1.04–1.20)**
Hypertension	471 (73.5)	325 (69.2)	0.12	1.06 (0.99–1.15)
Body mass index > 40	85 (81.0)	711 (70.7)	**0.030**	**1.14** **(1.02–1.25)**
P1	378 (80.4)	418 (65.2)	**< 0.0001**	**1.23** **(1.15–1.33)**
Fentanyl	418 (73.7)	378 (69.5)	0.13	1.06 (0.99–1.14)
Difficult airway	68 (70.1)	698 (71.7)	0.72	0.98 (0.84–1.10)

P1: orotracheal intubation performer with < 1 year of experience. Source: Elaborated by the authors.

## Discussion

The results presented here show that the use of ETO as a hypnotic for OTI in the emergency room is not associated with a significant difference in early morbidity or mortality, or mortality within 30 days of hospitalization, when compared with other hypnotics. These results are like those obtained by Upchurch et al.^10^, who demonstrated the use of both ETO and ketamine to perform rapid sequence intubation induction for adult trauma patients produced similar results. However, a recent retrospective study comparing various hypnotics in patients with COVID-19 requiring OTI found increased mortality in patients using ETO when compared to midazolam, thiopental, and ketamine^17^.

Changes in BP before and after orotracheal intubation are associated with early mortality of patients, as in this sample. The fact that ETO produces less marked BP depression has led many professionals to choose ETO in the emergency room. A randomized clinical trial showed that propofol caused reduction in mean arterial pressure than ETO in anesthesia induction for cardiac surgery^11^^–^ 13. However, another study compared ETO to ketamine/propofol and found no difference in the evolution of peri-intubation BP and serious adverse events, though the admission and intubation were earlier in the ETO group, which could mean that the most severe patients were intubated with ETO and thus mask some its putative benefits^18^.

ETO use has been also proposed to lead to adrenal insufficiency due to suppression of the β-17-hydroxylase, but the effect is preventable with methylprednisolone administration prior to procedure^11^^,^ 18. It is unclear if adrenal effects could have any impact on ETO-associated mortality in patients with severe disease states, such as sepsis. On the contrary, a recent study showed that ETO was potentially a better alternative for hemodynamic stability than ketamine in this group of patients^19^. A systematic review and meta-analysis did not find conclusive evidence that a single dose of ETO in septic patients increased mortality^20^, while another recent study concluded that there was a relationship between mortality and ETO use in patients with high-critical illness scores^21^.

During the COVID-19 pandemic, there was a considerable increase in demand for medical professionals in the emergency room, leading to recruitment of professionals with less experience in emergency care. A systematic review and meta-analysis demonstrated a relationship between lack of experience with airway access and mortality, but the study involved the pre-hospital and paramedical categories of care demonstrated that experience is associated with higher odds of OTI success^22^^,^ 23 ^,^ but our study is the first to correlate in-hospital procedure experience to mortality.

In the univariate analysis for mortality within 30 days, duration of mechanical ventilation was related to increased mortality^24^. Similar results had been suggested by an observational study using multiple regression with a Cox proportional hazards model, in which mortality increased with duration of stay in the emergency room greater than 7 hours, and this time was correlated with intubation time^25^. In our study, the average ventilation time in the emergency department was 20 hours, which was partly due to hospital overcrowding due to the pandemic period^26^.

The present study did not use masking in the use of drugs. This limitation may have influenced our results. Study physicians were allowed to select specific patients for ketamine or ETO for individualized reasons, and these patients were excluded from the study.

## Conclusion

The present study showed that there was no difference in mortality between patients intubated in the emergency room with ETO and those who received non-ETO hypnotics.

## Data Availability

Data will be available upon request.
